# 1,3-Bis(2,3,5,6-tetra­fluoro-4-iodo­phen­oxy)-2,2-bis­[(2,3,5,6-tetra­fluoro-4-iodo­phen­oxy)meth­yl]propane

**DOI:** 10.1107/S1600536813007605

**Published:** 2013-03-23

**Authors:** Gabriella Cavallo, Pierangelo Metrangolo, Tullio Pilati, Giuseppe Resnati, Giancarlo Terraneo, Maurizio Ursini

**Affiliations:** a NFMLab, Department of Chemistry, Materials and Chemical Engineering, "G. Natta", Politecnico di Milano, Via Mancinelli, 7, I-20131 Milano, Italy

## Abstract

In the crystal structure of the title compound, C_29_H_8_F_16_I_4_O_4_, short I⋯I and I⋯F contacts, which can be understood as halogen bonds (XBs), represent the strongest inter­molecular inter­actions, consistent with the presence of I and F atoms, and the absence of H atoms, at the periphery of the mol­ecule. In addition, π–π stacking inter­actions between tetra­fluoro­iodo­phenyl (TFIP) groups and five short F⋯F inter­actions are present.

## Related literature

The title compound is a robust halogen-bonding (XB) donor tecton in supra­molecular chemistry. For background to XB-based crystal engeneering, see: Guido *et al.* (2004 ▶, 2005 ▶); Lucassen *et al.* (2007 ▶); Metrangolo *et al.* (2007 ▶). For the synthesis, see: Caronna *et al.* (2004 ▶). For a description of the Cambridge Structural Database, see: Allen (2002 ▶).
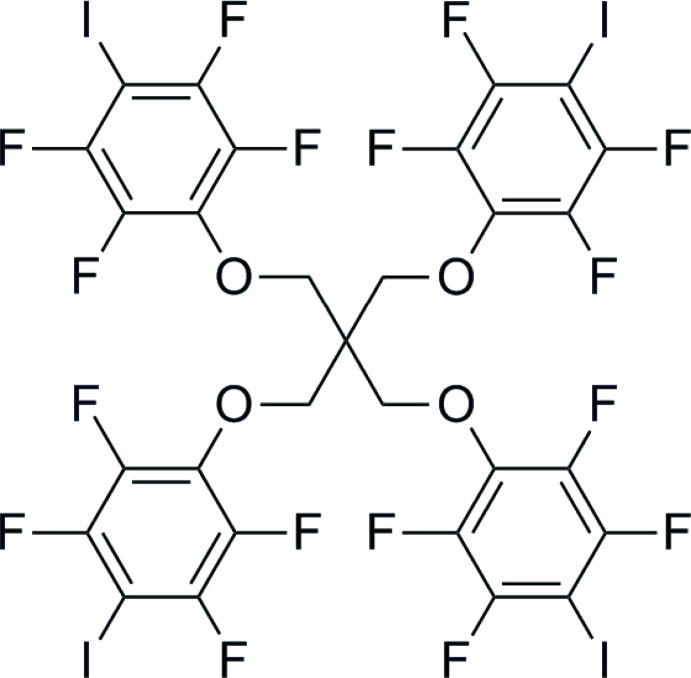


## Experimental

### 

#### Crystal data

C_29_H_8_F_16_I_4_O_4_
*M* *_r_* = 1231.95
Monoclinic, 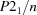

*a* = 7.9716 (9) Å
*b* = 20.665 (3) Å
*c* = 20.194 (4) Å
β = 92.745 (12)°
*V* = 3322.8 (9) Å^3^
*Z* = 4
Mo *K*α radiation
μ = 3.88 mm^−1^
*T* = 90 K
0.34 × 0.06 × 0.04 mm

#### Data collection

Bruker APEX 2000 CCD area-detector diffractometer
Absorption correction: multi-scan (*SADABS*; Bruker, 1998 ▶) *T*_min_ = 0.742, *T*_max_ = 1.000
39668 measured reflections
9728 independent reflections
7975 reflections with *I* > 2σ(*I*)
*R*_int_ = 0.042

#### Refinement

*R*[*F* ^2^ > 2σ(*F* ^2^)] = 0.038
*wR*(*F* ^2^) = 0.093
*S* = 1.04
9728 reflections
510 parameters
28 restraints
All H-atom parameters refined
Δρ_max_ = 2.08 e Å^−3^
Δρ_min_ = −0.54 e Å^−3^

### 

Data collection: *SMART* (Bruker, 1998 ▶); cell refinement: *SAINT* (Bruker, 1998 ▶); data reduction: *SAINT*; program(s) used to solve structure: *SIR2002* (Burla *et al.*, 2003 ▶); program(s) used to refine structure: *SHELXL2012* (Sheldrick, 2008 ▶); molecular graphics: *ORTEP-3 for Windows* (Farrugia, 2012 ▶) and *Mercury* (Macrae *et al.*, 2006 ▶); software used to prepare material for publication: *SHELXL2012*.

## Supplementary Material

Crystal structure: contains datablock(s) I, global. DOI: 10.1107/S1600536813007605/lh5584sup1.cif

Structure factors: contains datablock(s) I. DOI: 10.1107/S1600536813007605/lh5584Isup2.hkl

Supplementary material file. DOI: 10.1107/S1600536813007605/lh5584Isup3.cml

Additional supplementary materials:  crystallographic information; 3D view; checkCIF report

## Figures and Tables

**Table 1:** Halogen bonds and C—F⋯F—C and π–π short inter­actions (Å, °) The distances between the CNTRn of the four TFIP groups showing π–π inter­actions are also reported (CNTRn is the centroid of the benzene group linking the In iodine atom).

C—*X*⋯*Y*(—C)	*X*⋯*Y*	C—*X*⋯*Y*	*X*⋯*Y*—C
C5—I1⋯I3^i^	3.7838 (6)	169.59 (11)	
C12—I2⋯F15^ii^	3.323 (3)	174.72 (11)	
C19—I3⋯F2^iii^	3.176 (3)	162.73 (11)	
C26—I4⋯F6^iv^	3.240 (3)	136.47 (11)	
C3—F1⋯(F12—C21)^v^	2.610 (3)	162.3 (2)	165.1 (3)
C4—F2⋯(F4—C7)^v^	2.790 (4)	150.2 (2)	149.5 (3)
C17—F9⋯(F11—C20)^v^	2.771 (4)	151.1 (2)	150.6 (3)
C10—F5⋯(F7—C13)^v^	2.679 (3)	158.8 (2)	157.2 (3)
C25—F14⋯(F16—C28)^vi^	2.821 (3)	147.6 (2)	149.2 (3)
CNTR1⋯CNTR4^i^	3.643 (6)		
C3⋯C27^i^	3.334 (5)		
C16⋯C11^vii^	3.317 (5)		
C18⋯C9^vii^	3.307 (5)		
F9⋯C13^vii^	3.156 (5)		
CNTR2⋯CNTR3^vii^	3.648 (6)		

Symmetry codes: (i) 
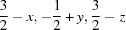
; (ii) 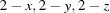; (iii) 
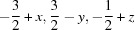
; (iv) 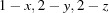; (v) 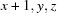; (vi) 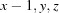; (vii) 
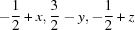
.

**Fig. 1.**
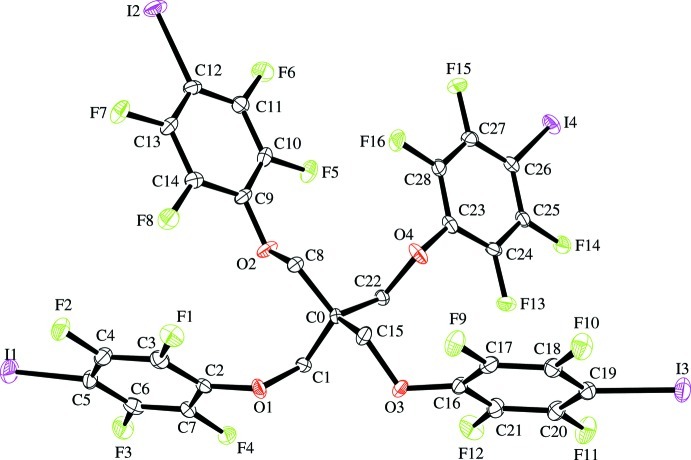
The molecular structure of the title compound with ellipsoids drawn at the 50% probability level.

**Fig. 2.**
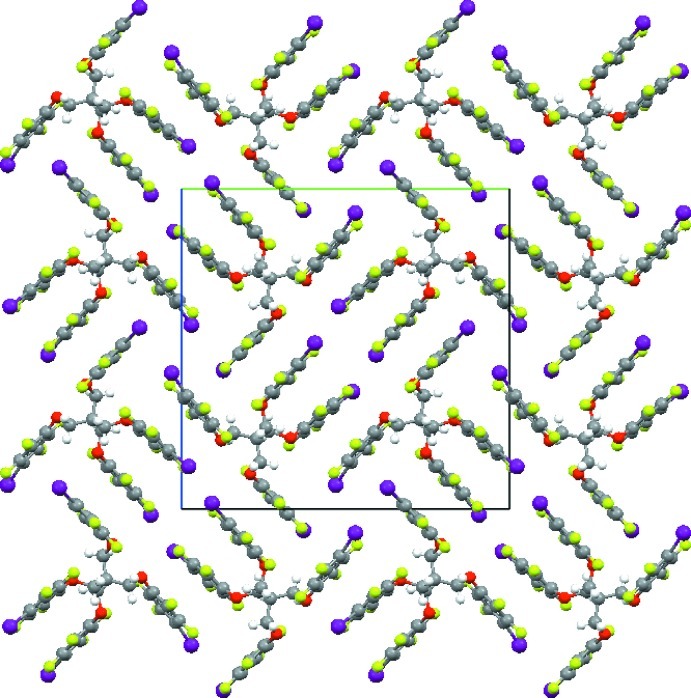
The crystal packing viewed along the a axis highlighting the π—π interactions between benzene rings.

## supplementary crystallographic information

### Comment

The title compound is a flexible molecule synthesized and utilized for halogen bonding (XB) based crystal engeneering (Guido *et al.*, 2004, 2005; Metrangolo *et al.*, 2007). Here we present the structure of the pure compound which is characterized by the presence of I···I and I···F XBs, short F···F contacts and π—π stacking interactions between couples of TFIP pendants. The C—I···F short contancts are particularly interesting as they show C—I···F angles consistent with bona fide XBs. The shortest C—I···F contanct is 3.176 (3) Å (see Table 1) and only few shorter distances are found in the Cambridge Structural Database (Version 5.33; Allen, 2002), the shortest being in WEXVUR (2.962 Å, Lucassen *et al.*, 2007). The molecular symmetry is approximately C_2_; the torsion angles C0—C1—O1—C2, C0—C8—O2—C9, C0—C15—O3—C16, C0—C22—O4—C23 are 120.9 (3), -162.0 (3), 118.0 (4) and -164.4 (3) °, respectively. Pertinent geometric data are listed in Table 1. The TFIP1 and TFIP3, the phenyl rings bearing I1 and I3, are nearly coplanar and anti-parallel; TFIP2 and TFIP4, the phenyl rings bearing I2 and I4, are not coplanar, but nearly parallel. This conformation favours the formation of π···π interactions between the couples TFIP1/TFIP4 and TFIP2/TFIP3. Supplementary Table A reports the main interactions of the structure. Figure 2 shows the crystal packing.

### Experimental

The synthesis of the compound was reported by Caronna *et al.*, (2004). Crystals for X-ray analysis were obtained *via* isothermal evaporation of a chloroform solution.

### Refinement

H atoms were obtained by difference map. They were refined independently with isotropic displacement parameters but restrained to have approximately the same C—H distances.

### Crystal data

**Table:**

C_29_H_8_F_16_I_4_O_4_	*F*(000) = 2280
*M**_r_* = 1231.95	*D*_x_ = 2.463 Mg m^−^^3^
Monoclinic, *P*2_1_/*n*	Mo *K*α radiation, λ = 0.71073 Å
*a* = 7.9716 (9) Å	Cell parameters from 8992 reflections
*b* = 20.665 (3) Å	θ = 2.2–30.0°
*c* = 20.194 (4) Å	µ = 3.88 mm^−^^1^
β = 92.745 (12)°	*T* = 90 K
*V* = 3322.8 (9) Å^3^	Neddle, colourless
*Z* = 4	0.34 × 0.06 × 0.04 mm

### Data collection

**Table:**

Bruker APEX 2000 CCD area-detector diffractometer	9728 independent reflections
Radiation source: fine-focus sealed tube	7975 reflections with *I* > 2σ(*I*)
Graphite monochromator	*R*_int_ = 0.042
φ and ω scans	θ_max_ = 30.1°, θ_min_ = 1.4°
Absorption correction: multi-scan (*SADABS*; Bruker, 1998)	*h* = −11→11
*T*_min_ = 0.742, *T*_max_ = 1.000	*k* = −29→25
39668 measured reflections	*l* = −28→28

### Refinement

**Table:**

Refinement on *F*^2^	Primary atom site location: structure-invariant direct methods
Least-squares matrix: full	Secondary atom site location: difference Fourier map
*R*[*F*^2^ > 2σ(*F*^2^)] = 0.038	Hydrogen site location: difference Fourier map
*wR*(*F*^2^) = 0.093	All H-atom parameters refined
*S* = 1.04	*w* = 1/[σ^2^(*F*_o_^2^) + (0.0456*P*)^2^ + 4.8626*P*] where *P* = (*F*_o_^2^ + 2*F*_c_^2^)/3
9728 reflections	(Δ/σ)_max_ = 0.004
510 parameters	Δρ_max_ = 2.08 e Å^−^^3^
28 restraints	Δρ_min_ = −0.54 e Å^−^^3^

### Special details

**Table:**

Experimental. OXFORD low temperature device.
Geometry. All e.s.d.'s (except the e.s.d. in the dihedral angle between two l.s. planes) are estimated using the full covariance matrix. The cell e.s.d.'s are taken into account individually in the estimation of e.s.d.'s in distances, angles and torsion angles; correlations between e.s.d.'s in cell parameters are only used when they are defined by crystal symmetry. An approximate (isotropic) treatment of cell e.s.d.'s is used for estimating e.s.d.'s involving l.s. planes.
Refinement. H atoms were restrained to have similar C—H distances.

### Fractional atomic coordinates and isotropic or equivalent isotropic displacement parameters (Å^2^)

**Table:**

	*x*	*y*	*z*	*U*_iso_*/*U*_eq_	
C0	0.6874 (4)	0.72962 (16)	0.72029 (16)	0.0128 (6)	
C1	0.6616 (4)	0.65897 (17)	0.73747 (18)	0.0154 (7)	
H1A	0.662 (5)	0.6502 (19)	0.7821 (10)	0.011 (10)*	
H1B	0.566 (4)	0.644 (2)	0.716 (2)	0.026 (12)*	
O1	0.7997 (3)	0.62098 (12)	0.71393 (12)	0.0184 (5)	
C2	0.8909 (5)	0.58713 (16)	0.76047 (17)	0.0152 (7)	
C3	1.0636 (5)	0.59535 (18)	0.76558 (17)	0.0183 (7)	
C4	1.1616 (5)	0.56147 (19)	0.81117 (18)	0.0201 (7)	
C5	1.0932 (5)	0.51753 (19)	0.85359 (17)	0.0198 (7)	
C6	0.9202 (5)	0.50767 (18)	0.84804 (18)	0.0207 (7)	
C7	0.8215 (5)	0.54139 (18)	0.80207 (18)	0.0175 (7)	
F1	1.1365 (3)	0.63721 (11)	0.72512 (11)	0.0240 (5)	
F2	1.3281 (3)	0.57269 (12)	0.81383 (12)	0.0256 (5)	
F3	0.8473 (3)	0.46500 (12)	0.88729 (12)	0.0293 (5)	
F4	0.6556 (3)	0.53013 (11)	0.79721 (12)	0.0224 (5)	
I1	1.24282 (4)	0.47021 (2)	0.92510 (2)	0.03126 (8)	
C8	0.8473 (4)	0.75684 (17)	0.75458 (16)	0.0135 (6)	
H8A	0.940 (4)	0.7406 (19)	0.7350 (18)	0.013 (10)*	
H8B	0.857 (5)	0.8008 (10)	0.7508 (19)	0.012 (10)*	
C9	0.9445 (4)	0.78098 (18)	0.86461 (16)	0.0157 (7)	
C10	0.8797 (4)	0.82522 (18)	0.90826 (17)	0.0171 (7)	
C11	0.9831 (5)	0.86081 (18)	0.95143 (17)	0.0176 (7)	
C12	1.1559 (5)	0.85353 (17)	0.95290 (16)	0.0170 (7)	
C13	1.2210 (5)	0.80928 (18)	0.90966 (17)	0.0173 (7)	
C14	1.1183 (5)	0.77370 (18)	0.86650 (17)	0.0168 (7)	
O2	0.8390 (3)	0.74463 (13)	0.82499 (12)	0.0192 (5)	
F5	0.7123 (3)	0.83272 (12)	0.90892 (11)	0.0241 (5)	
F6	0.9105 (3)	0.90175 (12)	0.99275 (12)	0.0279 (5)	
F7	1.3870 (3)	0.80009 (12)	0.90866 (11)	0.0249 (5)	
F8	1.1877 (3)	0.73036 (12)	0.82613 (11)	0.0239 (5)	
I2	1.31029 (4)	0.90660 (2)	1.01837 (2)	0.02597 (7)	
C15	0.7072 (5)	0.73904 (19)	0.64589 (17)	0.0161 (7)	
H15A	0.714 (5)	0.7818 (10)	0.6374 (19)	0.010 (9)*	
H15B	0.803 (4)	0.721 (2)	0.633 (2)	0.024 (12)*	
O3	0.5646 (3)	0.70967 (12)	0.60950 (12)	0.0186 (5)	
C16	0.4631 (4)	0.74785 (18)	0.57170 (16)	0.0157 (7)	
C17	0.5163 (4)	0.79214 (18)	0.52491 (17)	0.0164 (7)	
C18	0.4019 (5)	0.82563 (18)	0.48473 (16)	0.0166 (7)	
C19	0.2301 (5)	0.81733 (18)	0.48965 (17)	0.0175 (7)	
C20	0.1772 (4)	0.77314 (19)	0.53568 (18)	0.0179 (7)	
C21	0.2898 (5)	0.73955 (18)	0.57590 (17)	0.0182 (7)	
F9	0.6814 (3)	0.80003 (12)	0.51691 (11)	0.0226 (5)	
F10	0.4615 (3)	0.86711 (12)	0.44021 (11)	0.0256 (5)	
F11	0.0129 (3)	0.76437 (13)	0.54335 (12)	0.0295 (5)	
F12	0.2283 (3)	0.69714 (12)	0.61902 (11)	0.0278 (5)	
I3	0.05865 (3)	0.87228 (2)	0.43353 (2)	0.02431 (7)	
I4	0.07305 (3)	1.02190 (2)	0.86697 (2)	0.02284 (7)	
C22	0.5324 (4)	0.76548 (17)	0.74305 (17)	0.0145 (6)	
H22A	0.436 (3)	0.7464 (17)	0.7253 (18)	0.009 (9)*	
H22B	0.530 (5)	0.7641 (19)	0.7882 (10)	0.011 (10)*	
O4	0.5447 (3)	0.83249 (12)	0.72219 (13)	0.0195 (5)	
C23	0.4392 (5)	0.87347 (17)	0.75274 (18)	0.0163 (7)	
C24	0.2667 (5)	0.87194 (16)	0.74154 (18)	0.0161 (7)	
C25	0.1639 (4)	0.91486 (17)	0.77298 (18)	0.0161 (7)	
C26	0.2294 (5)	0.96073 (16)	0.81658 (17)	0.0156 (7)	
C27	0.4023 (5)	0.96312 (17)	0.82737 (18)	0.0177 (7)	
C28	0.5057 (5)	0.92034 (18)	0.79581 (18)	0.0181 (7)	
F13	0.1975 (3)	0.82806 (10)	0.69915 (11)	0.0214 (5)	
F14	−0.0017 (3)	0.91053 (11)	0.76005 (12)	0.0223 (5)	
F15	0.4729 (3)	1.00702 (11)	0.86817 (11)	0.0243 (5)	
F16	0.6725 (3)	0.92467 (12)	0.80682 (12)	0.0250 (5)	

### Atomic displacement parameters (Å^2^)

**Table:**

	*U*^11^	*U*^22^	*U*^33^	*U*^12^	*U*^13^	*U*^23^
C0	0.0116 (16)	0.0115 (15)	0.0148 (14)	0.0032 (12)	−0.0027 (12)	−0.0006 (12)
C1	0.0133 (17)	0.0122 (16)	0.0205 (16)	0.0006 (13)	−0.0013 (13)	−0.0027 (12)
O1	0.0207 (13)	0.0171 (13)	0.0174 (12)	0.0100 (10)	−0.0006 (10)	−0.0018 (9)
C2	0.0187 (17)	0.0102 (15)	0.0167 (15)	0.0028 (13)	−0.0003 (13)	−0.0027 (12)
C3	0.0203 (18)	0.0180 (18)	0.0168 (15)	0.0021 (14)	0.0032 (13)	−0.0026 (13)
C4	0.0168 (18)	0.0224 (19)	0.0208 (17)	0.0005 (15)	−0.0022 (14)	−0.0037 (14)
C5	0.0202 (19)	0.0208 (19)	0.0176 (16)	0.0060 (15)	−0.0059 (14)	−0.0017 (13)
C6	0.028 (2)	0.0130 (17)	0.0214 (17)	−0.0007 (15)	0.0034 (15)	0.0003 (13)
C7	0.0169 (17)	0.0145 (16)	0.0210 (16)	0.0040 (13)	−0.0009 (13)	−0.0038 (13)
F1	0.0238 (12)	0.0220 (12)	0.0269 (11)	−0.0029 (9)	0.0090 (9)	0.0019 (9)
F2	0.0147 (11)	0.0311 (13)	0.0305 (12)	0.0014 (9)	−0.0038 (9)	−0.0026 (10)
F3	0.0316 (14)	0.0233 (12)	0.0329 (13)	−0.0021 (10)	−0.0005 (10)	0.0100 (10)
F4	0.0168 (11)	0.0186 (11)	0.0312 (12)	−0.0034 (9)	−0.0031 (9)	0.0004 (9)
I1	0.03263 (16)	0.02794 (15)	0.03187 (14)	0.00387 (11)	−0.01244 (11)	0.00821 (11)
C8	0.0131 (16)	0.0148 (16)	0.0125 (14)	−0.0018 (13)	−0.0006 (12)	0.0000 (12)
C9	0.0155 (17)	0.0180 (17)	0.0133 (14)	−0.0061 (13)	−0.0038 (12)	0.0021 (12)
C10	0.0129 (17)	0.0216 (18)	0.0167 (15)	−0.0009 (14)	−0.0011 (12)	0.0056 (13)
C11	0.0226 (19)	0.0170 (17)	0.0131 (14)	0.0009 (14)	−0.0006 (13)	−0.0003 (12)
C12	0.0221 (18)	0.0147 (16)	0.0139 (15)	−0.0053 (14)	−0.0041 (13)	0.0009 (12)
C13	0.0143 (17)	0.0223 (18)	0.0150 (15)	−0.0040 (14)	−0.0028 (12)	0.0011 (13)
C14	0.0186 (18)	0.0164 (17)	0.0154 (15)	−0.0024 (14)	0.0002 (13)	−0.0005 (12)
O2	0.0203 (13)	0.0232 (14)	0.0136 (11)	−0.0096 (11)	−0.0045 (9)	0.0005 (10)
F5	0.0120 (10)	0.0334 (13)	0.0269 (11)	0.0013 (9)	0.0000 (9)	0.0007 (10)
F6	0.0315 (14)	0.0274 (13)	0.0248 (11)	0.0041 (10)	0.0015 (10)	−0.0110 (10)
F7	0.0136 (11)	0.0354 (14)	0.0254 (11)	−0.0012 (10)	−0.0026 (9)	−0.0015 (10)
F8	0.0204 (12)	0.0301 (13)	0.0213 (10)	0.0025 (10)	0.0017 (9)	−0.0077 (9)
I2	0.03197 (15)	0.02485 (14)	0.02000 (12)	−0.01119 (11)	−0.01007 (10)	−0.00024 (9)
C15	0.0126 (16)	0.0210 (18)	0.0142 (15)	0.0009 (14)	−0.0030 (12)	−0.0007 (13)
O3	0.0176 (13)	0.0178 (13)	0.0195 (12)	−0.0019 (10)	−0.0090 (10)	0.0009 (10)
C16	0.0159 (17)	0.0174 (17)	0.0135 (14)	0.0017 (13)	−0.0031 (12)	−0.0017 (12)
C17	0.0142 (17)	0.0191 (17)	0.0157 (15)	−0.0009 (13)	−0.0006 (12)	−0.0040 (13)
C18	0.0190 (18)	0.0176 (17)	0.0133 (14)	0.0002 (14)	0.0006 (13)	0.0009 (12)
C19	0.0176 (17)	0.0168 (17)	0.0179 (15)	0.0031 (14)	−0.0018 (13)	−0.0022 (13)
C20	0.0113 (16)	0.0228 (19)	0.0197 (16)	−0.0003 (14)	0.0007 (13)	−0.0005 (14)
C21	0.0210 (18)	0.0195 (18)	0.0139 (15)	−0.0001 (14)	0.0002 (13)	0.0036 (13)
F9	0.0127 (10)	0.0333 (13)	0.0217 (10)	0.0002 (9)	0.0018 (8)	0.0030 (9)
F10	0.0238 (12)	0.0287 (13)	0.0243 (11)	−0.0037 (10)	0.0005 (9)	0.0107 (9)
F11	0.0133 (11)	0.0420 (15)	0.0328 (13)	−0.0044 (10)	−0.0010 (9)	0.0077 (11)
F12	0.0380 (14)	0.0234 (12)	0.0228 (11)	−0.0044 (10)	0.0108 (10)	0.0116 (9)
I3	0.02068 (13)	0.02714 (14)	0.02442 (12)	0.00478 (10)	−0.00593 (9)	0.00477 (10)
I4	0.03133 (15)	0.01636 (12)	0.02106 (12)	0.00833 (10)	0.00373 (10)	−0.00080 (9)
C22	0.0116 (16)	0.0122 (16)	0.0196 (16)	0.0016 (12)	−0.0002 (12)	−0.0007 (12)
O4	0.0197 (13)	0.0116 (12)	0.0280 (13)	0.0049 (10)	0.0093 (11)	0.0035 (10)
C23	0.0158 (17)	0.0118 (16)	0.0217 (16)	0.0030 (13)	0.0033 (13)	0.0030 (13)
C24	0.0168 (17)	0.0092 (15)	0.0222 (16)	−0.0006 (13)	0.0007 (13)	−0.0015 (12)
C25	0.0131 (16)	0.0116 (16)	0.0237 (17)	0.0017 (13)	0.0008 (13)	0.0024 (13)
C26	0.0195 (18)	0.0105 (15)	0.0170 (15)	0.0018 (13)	0.0025 (13)	0.0018 (12)
C27	0.0209 (18)	0.0138 (16)	0.0181 (16)	−0.0034 (14)	−0.0021 (13)	0.0018 (13)
C28	0.0157 (17)	0.0134 (16)	0.0247 (17)	−0.0003 (13)	−0.0035 (14)	0.0049 (13)
F13	0.0207 (11)	0.0154 (11)	0.0277 (11)	0.0003 (9)	−0.0019 (9)	−0.0069 (9)
F14	0.0136 (11)	0.0179 (11)	0.0352 (12)	0.0022 (9)	0.0002 (9)	−0.0023 (9)
F15	0.0289 (13)	0.0201 (11)	0.0229 (11)	−0.0039 (10)	−0.0082 (9)	−0.0039 (9)
F16	0.0112 (11)	0.0255 (12)	0.0377 (13)	−0.0007 (9)	−0.0045 (9)	0.0058 (10)

### Geometric parameters (Å, º)

**Table:**

C0—C1	1.517 (5)	C13—C14	1.379 (5)
C0—C8	1.528 (5)	C14—F8	1.348 (4)
C0—C15	1.531 (5)	C15—O3	1.456 (4)
C0—C22	1.531 (5)	C15—H15A	0.903 (19)
C1—O1	1.451 (4)	C15—H15B	0.91 (2)
C1—H1A	0.919 (19)	O3—C16	1.342 (4)
C1—H1B	0.91 (2)	C16—C17	1.396 (5)
O1—C2	1.355 (4)	C16—C21	1.399 (5)
C2—C3	1.386 (5)	C17—F9	1.344 (4)
C2—C7	1.396 (5)	C17—C18	1.378 (5)
C3—F1	1.341 (4)	C18—F10	1.345 (4)
C3—C4	1.371 (5)	C18—C19	1.389 (5)
C4—F2	1.346 (4)	C19—C20	1.383 (5)
C4—C5	1.379 (5)	C19—I3	2.072 (4)
C5—C6	1.393 (6)	C20—F11	1.339 (4)
C5—I1	2.073 (4)	C20—C21	1.370 (5)
C6—F3	1.337 (4)	C21—F12	1.345 (4)
C6—C7	1.377 (5)	I4—C26	2.076 (3)
C7—F4	1.341 (4)	C22—O4	1.452 (4)
C8—O2	1.449 (4)	C22—H22A	0.920 (19)
C8—H8A	0.920 (19)	C22—H22B	0.913 (19)
C8—H8B	0.915 (19)	O4—C23	1.362 (4)
C9—O2	1.359 (4)	C23—C24	1.383 (5)
C9—C10	1.387 (5)	C23—C28	1.390 (5)
C9—C14	1.392 (5)	C24—F13	1.347 (4)
C10—F5	1.344 (4)	C24—C25	1.383 (5)
C10—C11	1.382 (5)	C25—F14	1.337 (4)
C11—F6	1.339 (4)	C25—C26	1.379 (5)
C11—C12	1.385 (5)	C26—C27	1.385 (5)
C12—C13	1.382 (5)	C27—F15	1.332 (4)
C12—I2	2.076 (3)	C27—C28	1.385 (5)
C13—F7	1.338 (4)	C28—F16	1.340 (4)
			
C1—C0—C8	111.7 (3)	F8—C14—C9	119.5 (3)
C1—C0—C15	111.6 (3)	C13—C14—C9	121.3 (3)
C8—C0—C15	106.0 (3)	C9—O2—C8	115.2 (3)
C1—C0—C22	106.1 (3)	O3—C15—C0	109.1 (3)
C8—C0—C22	110.7 (3)	O3—C15—H15A	112 (3)
C15—C0—C22	110.8 (3)	C0—C15—H15A	109 (2)
O1—C1—C0	109.4 (3)	O3—C15—H15B	109 (3)
O1—C1—H1A	104 (3)	C0—C15—H15B	111 (3)
C0—C1—H1A	115 (3)	H15A—C15—H15B	107 (4)
O1—C1—H1B	107 (3)	C16—O3—C15	118.5 (3)
C0—C1—H1B	109 (3)	O3—C16—C17	125.2 (3)
H1A—C1—H1B	112 (4)	O3—C16—C21	117.6 (3)
C2—O1—C1	116.3 (3)	C17—C16—C21	117.0 (3)
O1—C2—C3	119.1 (3)	F9—C17—C18	119.5 (3)
O1—C2—C7	123.5 (3)	F9—C17—C16	119.5 (3)
C3—C2—C7	117.3 (3)	C18—C17—C16	120.9 (3)
F1—C3—C4	119.2 (3)	F10—C18—C17	118.0 (3)
F1—C3—C2	119.5 (3)	F10—C18—C19	120.5 (3)
C4—C3—C2	121.3 (3)	C17—C18—C19	121.5 (3)
F2—C4—C3	117.9 (3)	C20—C19—C18	117.6 (3)
F2—C4—C5	120.5 (3)	C20—C19—I3	121.0 (3)
C3—C4—C5	121.6 (4)	C18—C19—I3	121.3 (3)
C4—C5—C6	117.8 (3)	F11—C20—C21	118.7 (3)
C4—C5—I1	120.8 (3)	F11—C20—C19	119.9 (3)
C6—C5—I1	121.4 (3)	C21—C20—C19	121.4 (3)
F3—C6—C7	118.9 (4)	F12—C21—C20	117.7 (3)
F3—C6—C5	120.3 (3)	F12—C21—C16	120.8 (3)
C7—C6—C5	120.8 (3)	C20—C21—C16	121.5 (3)
F4—C7—C6	119.4 (3)	O4—C22—C0	107.9 (3)
F4—C7—C2	119.4 (3)	O4—C22—H22A	111 (2)
C6—C7—C2	121.2 (4)	C0—C22—H22A	110 (2)
O2—C8—C0	107.7 (3)	O4—C22—H22B	109 (3)
O2—C8—H8A	116 (3)	C0—C22—H22B	110 (3)
C0—C8—H8A	110 (3)	H22A—C22—H22B	108 (3)
O2—C8—H8B	105 (2)	C23—O4—C22	114.2 (3)
C0—C8—H8B	113 (3)	O4—C23—C24	122.8 (3)
H8A—C8—H8B	105 (4)	O4—C23—C28	119.5 (3)
O2—C9—C10	120.0 (3)	C24—C23—C28	117.7 (3)
O2—C9—C14	122.9 (3)	F13—C24—C25	119.3 (3)
C10—C9—C14	117.0 (3)	F13—C24—C23	119.6 (3)
F5—C10—C11	119.6 (3)	C25—C24—C23	121.1 (3)
F5—C10—C9	118.9 (3)	F14—C25—C26	120.7 (3)
C11—C10—C9	121.5 (3)	F14—C25—C24	118.0 (3)
F6—C11—C10	117.8 (3)	C26—C25—C24	121.3 (3)
F6—C11—C12	121.0 (3)	C25—C26—C27	117.9 (3)
C10—C11—C12	121.2 (3)	C25—C26—I4	120.9 (3)
C13—C12—C11	117.5 (3)	C27—C26—I4	121.2 (3)
C13—C12—I2	121.5 (3)	F15—C27—C28	118.4 (3)
C11—C12—I2	121.0 (3)	F15—C27—C26	120.6 (3)
F7—C13—C14	118.2 (3)	C28—C27—C26	121.0 (3)
F7—C13—C12	120.3 (3)	F16—C28—C27	119.3 (3)
C14—C13—C12	121.5 (3)	F16—C28—C23	119.7 (3)
F8—C14—C13	119.2 (3)	C27—C28—C23	121.0 (3)
			
C8—C0—C1—O1	−62.5 (3)	C1—C0—C15—O3	53.9 (4)
C15—C0—C1—O1	56.0 (4)	C8—C0—C15—O3	175.7 (3)
C22—C0—C1—O1	176.8 (3)	C22—C0—C15—O3	−64.1 (4)
C0—C1—O1—C2	120.9 (3)	C0—C15—O3—C16	118.0 (3)
C1—O1—C2—C3	−126.0 (3)	C15—O3—C16—C17	50.0 (5)
C1—O1—C2—C7	57.3 (4)	C15—O3—C16—C21	−134.8 (3)
O1—C2—C3—F1	1.0 (5)	O3—C16—C17—F9	−2.1 (5)
C7—C2—C3—F1	177.8 (3)	C21—C16—C17—F9	−177.3 (3)
O1—C2—C3—C4	−179.0 (3)	O3—C16—C17—C18	174.9 (3)
C7—C2—C3—C4	−2.1 (5)	C21—C16—C17—C18	−0.3 (5)
F1—C3—C4—F2	1.0 (5)	F9—C17—C18—F10	−2.4 (5)
C2—C3—C4—F2	−179.1 (3)	C16—C17—C18—F10	−179.3 (3)
F1—C3—C4—C5	−179.5 (3)	F9—C17—C18—C19	177.7 (3)
C2—C3—C4—C5	0.4 (6)	C16—C17—C18—C19	0.8 (5)
F2—C4—C5—C6	−179.5 (3)	F10—C18—C19—C20	178.9 (3)
C3—C4—C5—C6	1.0 (6)	C17—C18—C19—C20	−1.2 (5)
F2—C4—C5—I1	2.5 (5)	F10—C18—C19—I3	−4.0 (5)
C3—C4—C5—I1	−177.0 (3)	C17—C18—C19—I3	175.8 (3)
C4—C5—C6—F3	179.1 (3)	C18—C19—C20—F11	178.6 (3)
I1—C5—C6—F3	−2.9 (5)	I3—C19—C20—F11	1.5 (5)
C4—C5—C6—C7	−0.7 (6)	C18—C19—C20—C21	1.2 (5)
I1—C5—C6—C7	177.4 (3)	I3—C19—C20—C21	−175.9 (3)
F3—C6—C7—F4	−0.8 (5)	F11—C20—C21—F12	3.0 (5)
C5—C6—C7—F4	179.0 (3)	C19—C20—C21—F12	−179.5 (3)
F3—C6—C7—C2	179.1 (3)	F11—C20—C21—C16	−178.2 (3)
C5—C6—C7—C2	−1.1 (6)	C19—C20—C21—C16	−0.8 (6)
O1—C2—C7—F4	−0.9 (5)	O3—C16—C21—F12	3.5 (5)
C3—C2—C7—F4	−177.6 (3)	C17—C16—C21—F12	179.1 (3)
O1—C2—C7—C6	179.2 (3)	O3—C16—C21—C20	−175.3 (3)
C3—C2—C7—C6	2.4 (5)	C17—C16—C21—C20	0.3 (5)
C1—C0—C8—O2	−54.2 (4)	C1—C0—C22—O4	−175.0 (3)
C15—C0—C8—O2	−176.0 (3)	C8—C0—C22—O4	63.6 (3)
C22—C0—C8—O2	63.8 (3)	C15—C0—C22—O4	−53.6 (4)
O2—C9—C10—F5	−2.1 (5)	C0—C22—O4—C23	−164.4 (3)
C14—C9—C10—F5	−178.7 (3)	C22—O4—C23—C24	−68.8 (4)
O2—C9—C10—C11	177.0 (3)	C22—O4—C23—C28	113.0 (4)
C14—C9—C10—C11	0.4 (5)	O4—C23—C24—F13	0.5 (5)
F5—C10—C11—F6	0.1 (5)	C28—C23—C24—F13	178.7 (3)
C9—C10—C11—F6	−178.9 (3)	O4—C23—C24—C25	−179.3 (3)
F5—C10—C11—C12	178.9 (3)	C28—C23—C24—C25	−1.1 (5)
C9—C10—C11—C12	−0.2 (5)	F13—C24—C25—F14	0.4 (5)
F6—C11—C12—C13	178.6 (3)	C23—C24—C25—F14	−179.8 (3)
C10—C11—C12—C13	−0.1 (5)	F13—C24—C25—C26	−179.8 (3)
F6—C11—C12—I2	−0.8 (5)	C23—C24—C25—C26	0.0 (5)
C10—C11—C12—I2	−179.5 (3)	F14—C25—C26—C27	−179.4 (3)
C11—C12—C13—F7	179.9 (3)	C24—C25—C26—C27	0.8 (5)
I2—C12—C13—F7	−0.6 (5)	F14—C25—C26—I4	2.8 (5)
C11—C12—C13—C14	0.1 (5)	C24—C25—C26—I4	−177.0 (3)
I2—C12—C13—C14	179.5 (3)	C25—C26—C27—F15	179.0 (3)
F7—C13—C14—F8	1.3 (5)	I4—C26—C27—F15	−3.2 (5)
C12—C13—C14—F8	−178.9 (3)	C25—C26—C27—C28	−0.6 (5)
F7—C13—C14—C9	−179.7 (3)	I4—C26—C27—C28	177.2 (3)
C12—C13—C14—C9	0.1 (6)	F15—C27—C28—F16	−0.5 (5)
O2—C9—C14—F8	2.2 (5)	C26—C27—C28—F16	179.1 (3)
C10—C9—C14—F8	178.6 (3)	F15—C27—C28—C23	180.0 (3)
O2—C9—C14—C13	−176.8 (3)	C26—C27—C28—C23	−0.5 (5)
C10—C9—C14—C13	−0.4 (5)	O4—C23—C28—F16	0.0 (5)
C10—C9—O2—C8	115.1 (4)	C24—C23—C28—F16	−178.3 (3)
C14—C9—O2—C8	−68.6 (4)	O4—C23—C28—C27	179.5 (3)
C0—C8—O2—C9	−162.0 (3)	C24—C23—C28—C27	1.3 (5)

### Halogen bonds and C—F···F—C and π–π short interactions (Å, °)

The distances between the CNTRn of the four TFIP groups showing π–π interactions are also reported (CNTRn is the centroid of the benzene group linking the In iodine atom).

**Table:**

C—*X*···*Y*(—C)	*X*···*Y*	C—*X*···*Y*	*X*···*Y*—C
C5—I1···I3^i^	3.7838 (6)	169.59 (11)	
C12—I2···F15^ii^	3.323 (3)	174.72 (11)	
C19—I3···F2^iii^	3.176 (3)	162.73 (11)	
C26—I4···F6^iv^	3.240 (3)	136.47 (11)	
C3—F1···(F12—C21)^v^	2.610 (3)	162.3 (2)	165.1 (3)
C4—F2···(F4—C7)^v^	2.790 (4)	150.2 (2)	149.5 (3)
C17—F9···(F11—C20)^v^	2.771 (4)	151.1 (2)	150.6 (3)
C10—F5···(F7—C13)^v^	2.679 (3)	158.8 (2)	157.2 (3)
C25—F14···(F16—C28)^vi^	2.821 (3)	147.6 (2)	149.2 (3)
CNTR1···CNTR4^i^	3.643 (6)		
C3···C27^i^	3.334 (5)		
C16···C11^vii^	3.317 (5)		
C18···C9^vii^	3.307 (5)		
F9···C13^vii^	3.156 (5)		
CNTR2···CNTR3^vii^	3.648 (6)		

Symmetry codes: (i) 3/2-x, -1/2+y, 3/2-z; (ii) 2-x, 2-y,2-z; (iii) -3/2+x, 3/2-y, -1/2+z; (iv) 1-x, 2-y, 2-z; (v) x+1,y,z; (vi) x-1,y,z; (vii) -1/2+x, 3/2-y, -1/2+z.
